# Comparison of the effect of consuming the prepared cakes with acorn flour and wheat flour following a hypocaloric diet on serum levels of leptin, endothelin, inflammatory factors, and oxidative stress parameters in obese and overweight patients with metabolic syndrome: A double‐blind clinical trial

**DOI:** 10.1002/fsn3.4393

**Published:** 2024-08-20

**Authors:** Hamed Sadeghi‐Dehsahraei, Siavash Babajafari, Mahboobeh Ashrafi, Mohsen Mohammadi‐Sartang

**Affiliations:** ^1^ Department of Basic Sciences, Faculty of Veterinary Medicine Shiraz University Shiraz Iran; ^2^ Nutrition Research Center, School of Nutrition and Food Science Shiraz University of Medical Sciences Shiraz Iran

**Keywords:** acorn, metabolic syndrome, MetS, obesity

## Abstract

Metabolic syndrome (MetS), which is a major consequence of obesity, increases mortality risks. Evidence shows favorable effects of nutritional approaches in the management of MetS. Accordingly, the use of functional foods has increased to enhance weight loss and reduce the risk factors associated with MetS. So, we aimed to investigate the effects of daily consumption of a functional acorn‐based cake in conjunction with energy‐restricted diet on some complications of patients with MetS. The study included 66 participants who were randomly assigned to either (A) a calorie‐restricted diet + functional cake (FC) (*n* = 33) or (B) a calorie‐restricted diet + a placebo cake (PC) (*n* = 33). Sociodemographic information, anthropometric measurements, dietary intakes, and serum biochemical parameters (inflammatory and oxidative stress markers, leptin, and endothelin) were measured before and after 8 weeks of intervention. Sixty‐three participants completed this trial. After adjustment for baseline levels, consumption of FC compared to the PC resulted in a significant decrease in IL‐6 (*p* = .03) and high‐sensitivity C‐reactive protein (*p* = .04) levels. No differences were observed between groups with regard to serum malondialdehyde, total antioxidant capacity, endothelin, and leptin levels (*p* > .05). Acorn‐based cake could improve inflammation as an adjunct to an energy‐restricted diet in overweight and obese patients with MetS. However, it is not clear whether acorn‐based cake can be used to prevent or treat MetS because of indecisive findings regarding its ability to manage oxidative stress and serum hormones.

## Graphical abstract

This study investigated the effect of consuming prepared cakes with acorn flour and wheat flour following a hypocaloric diet on serum levels of leptin, endothelin, inflammatory factors, and oxidative stress parameters in obese and overweight patients with metabolic syndrome in Shiraz province of Iran. It showed that acorn‐based cake could improve inflammation as an adjunct to an energy‐restricted diet in overweight and obese patients with MetS.

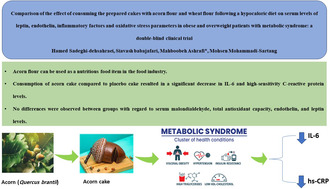

## 1 INTRODUCTION

One of the major consequences of obesity is metabolic syndrome (MetS), which increases the risk of cardiovascular disease, diabetes, and mortality (Kaur, 2014). The syndrome is a combination of chronic disease risk factors, including abdominal obesity (men >102 cm and women >88 cm), hypertension (≥130/≥85 mmHg), dyslipidemia (TG ≥1.7 mmol/L, HDL‐C; men <1.04 mmol/L and women <1.30 mmol/L), and impaired glucose tolerance (≥6.1 mmol/L) (Grundy, 2005). There is no clear explanation of how MetS develops; however, it has been suggested that insulin resistance (IR) in adipose tissue affects insulin‐mediated lipolysis inhibition and leads to higher circulating free fatty acids (FFAs) levels. FFAs in the blood inhibit the antilipolytic actions of insulin (Gallagher et al., 2010). The IR also increases serum viscosity, induces a prothrombotic state, and releases pro‐inflammatory cytokines from adipose tissue, which leads to an increased risk of cardio‐metabolic complications (Juhan‐Vague et al., 2003). Moreover, IR increases the production of Ang II which in turn increases the production of reactive oxygen species (ROS) (Mehta & Griendling, 2007; Vaněčková et al., 2014). Overproduction of ROS increases risk factors for MetS through the initiation of inflammation, impairment of endothelial cells, and proliferation of fibroblasts (Dai et al., 2013; Gobal et al., 2011). In previous research, there has been a link between the MetS and increased leptin (Esteghamati et al., 2011; Yun et al., 2010) and endothelin levels (Rocha et al., 2014). Interestingly, the evidence suggests that high serum leptin levels can be a predictor for MetS (Ghadge & Khaire, 2019). It is, therefore, critical to apply effective treatment strategies to improve inflammation, oxidative stress, and reduce leptin and endothelin hormone levels in the management of MetS.

Among the various treatment strategies, weight loss plays a major role in improving insulin sensitivity in MetS patients (Grundy et al., 2005). Currently, the use of functional foods has increased in conjunction with weight loss diets to increase weight loss and reduce the risk factors associated with MetS (Brown et al., 2015; Rebello et al., 2014). Accordingly, identifying functional foods that may assist energy‐restricted diets in facilitating positive results is an emerging area of research (Halford & Harrold, 2012). Historically, A large number of medicinal plants are naturally grown in different regions (Petrovska, 2012), especially in Iran. The Quercus genus (Fagaceae) contains 500 species; *Q brantii L* is the predominant species in the central and northern parts of Iran (Saffarzadeh et al., 1999). There is evidence that the fruit of an oak tree (Quercus tree), the acorn, contains notable vitamins and nutrients along with carbohydrates (Saffarzadeh et al., 1999). It also contains significant amounts of phenolics, tannins, catechins, epicatechins, and gallocatechins (Popović et al., 2013; Saffarzadeh et al., 1999).

The genus oak has been used for decades as an effective therapy for diseases such as chronic dermatological diseases, eczema, various (Aslani et al., 2009; Bahmani et al., 2015) microbial and viral diseases (Andrenšek et al., 2004; Basri & Fan, 2005; Muliawan et al., 2006), and gastrointestinal discomforts (Gharzouli et al., 1999). Recently, in vitro and in vivo studies of chronic diseases have demonstrated the antioxidation, antiproliferative, and anti‐inflammatory effects of various acorn components (Alizade Naini et al., 2021; Dogan et al., 2015; Moradi et al., 2016). Additionally, an animal study found that ellagitannins from *Quercus Petraea L* improved MetS signs and showed cardioprotective and hepatoprotective properties (Panchal & Brown, 2013). Nevertheless, human studies investigating the effects of acorns or acorn products on chronic diseases are limited (Sasani et al., 2023). Therefore, this randomized double‐blinded controlled trial (RCT) aimed to compare the effects of acorn flour cake with wheat flour cake following a hypocaloric diet on serum levels of leptin, endothelin, inflammatory factors, and oxidative stress parameters in obese and overweight patients with MetS.

## 2 METHODS AND PARTICIPANTS

### 2.1 Participants

**FIGURE 1:**
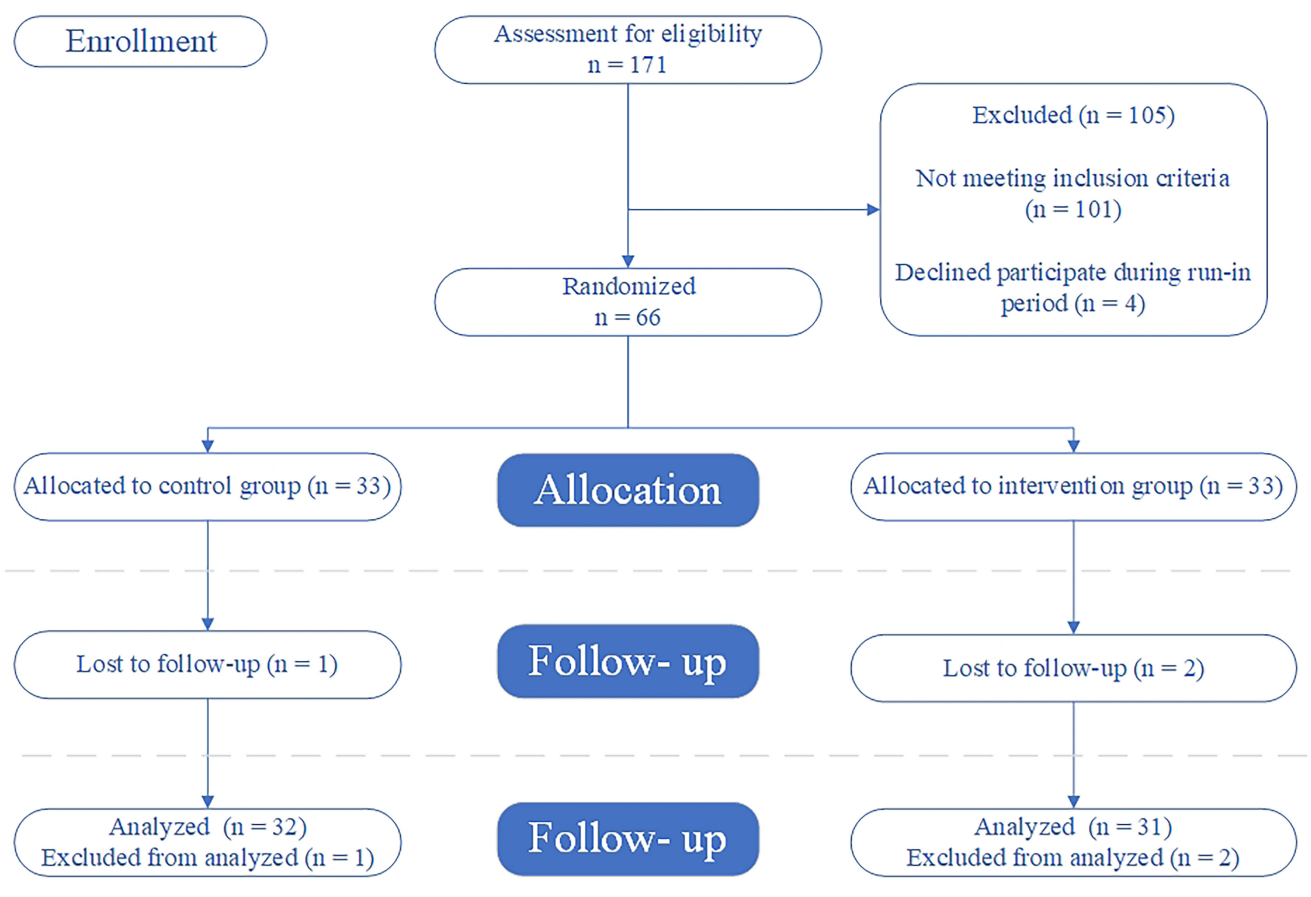
Flowchart of the study.

In this trial, a total of 66 participants were recruited from two clinics (Motahari and Imam Reza) affiliated with Shiraz University of Medical Sciences, Iran. Briefly, in a general announcement, 171 overweight and obese participants were enrolled, and after screening for inclusion criteria 70 eligible participants were entered into a 2‐week run‐in period. During the run‐in period, their dietary intake, physical activity level, and medical history were evaluated. For example, they were asked to record their dietary intakes for three nonconsecutive days (2 weekdays and 1 weekend day). Of them, four participants declined to participate and 66 participants were randomized into either the placebo cake (PC) or the functional cake (FC) groups (*n* = 33). (Figure 1). Subjects were included in the study if they were men and women between 20 and 60 years of age, had body mass index (BMI) of 25–35 kg/m^2^, and were diagnosed with MetS based on the National Cholesterol Education Program Adult Treatment Panel III criteria (Detection & Adults, 2002). They were also not included if they received drugs or supplements that could affect appetite, body weight, blood glucose, and lipid metabolism, received drugs or supplements with anti‐inflammatory effect, were pregnant and lactating women, had history of chronic disease (diabetes, cancer, renal, pulmonary, and hepatic diseases), had history of alcohol consumption, and lost more than 10% body weight within 6 months before the study initiation. More details about cake preparation have already been reported (Mohammadi‐Sartang et al., 2023). Accordingly, shell and internal layer (in Persian: Jaft) of collected dried acorn, *Q. brantii Lindl*, were removed and thereupon acorns were soaked in water for about 48 h to reduce astringent taste. Then, the fruits were dried and milled. After this, an amount of 10 g of treated acorn flour, whole egg, stevia, low‐fat milk, canola oil, baking powder, emulsifier, and vanilla essence was used in preparing each FC. All ingredients were similar and equal in amount for both case and control cakes except flour. The Nul flour (wheat flour without bran) was used in the control PC instead of acorn flour. Given that the calorie of treated acorn flour was more than Nul flour, we applied about 12 g Nul flour to make the calories of both cakes equal (each cake weighed about 30–35 g). Due to the natural brown color of acorn cakes, brown food color was used to make the cakes visually similar. Finally, basic chemical composition of the treated acorn flour and cakes including protein, fiber, ash, and moisture were determined using standard methods (Cunniff & Washington, 1997). Total carbohydrate contents were estimated by subtracting sum of fat, protein, ash, and moisture from 100%. Tannins of FC were measured by titration method and application of indigo solution as an identifier (WHO, 2003). The energy values of FC cakes were evaluated based on Atwater coefficients (carbohydrates and protein 4 kcal/g, fat 9 kcal/g) (Maclean et al., 2003). All the analyses were performed two times. In our study, the participants were intervened just one time in the afternoon.

Research protocol approval was obtained from the ethics committee at Shiraz University, Shiraz, Iran (IR.us.rec.1402.009), and registration was made in the Iranian Registry of Clinical Trials (IRCT20231104059958N1). An informed consent form was read and signed by all participants before they enrolled in the study.

### 2.2 Study design

After block randomization, study participants were randomly assigned to receive two daily servings (30 g) of FC (*n* = 33) or PC (*n* = 33) as snacks for 8 weeks. To maintain the blindness of the investigators and participants, the products were provided in identical packages. The placebos were designed in the same colors, sizes, and models as the intervention group. For blinding, the packs of cakes were coded in A and B by a third party who was not involved in the research process. Each 2‐week pack of FC and PC was prepared, and participants were checked for compliance as well as given their cakes to eat. Also, a table was designed and given to the participants to record their cake consumption. Moreover, the consumption of cakes was reminded via WhatsApp with a short message every day. We considered participants adherent if they consumed at least 90% of the cake products, and nonadherent if they missed eating more than 10% of the cake products. We carefully examined any potential side effects during visits, and those who complained of serious adverse effects were excluded from the study.

During the study, food consumption and energy intake were assessed via a 3‐day food recall (two weekdays and one weekend day). Then, nutritional values were analyzed using Nutritionist IV software (First Databank, San Bruno, CA, USA) modified for Iranian foods. At baseline, an equation recommended for overweight and obese subjects ≥19 years old was used to calculate total energy expenditure (TEE) (Mahan, 2016). Accordingly, an energy‐restricted diet composed of 55% carbohydrate, 30% fat, and 15% protein was given to all participants in each study group for 10 weeks (500 kcal fewer than the TEE). For better understanding, an exchange list was also given to participants.

### 2.3 Sociodemographic, anthropometric, and dietary intakes measurements

Demographic information of the participants was gathered using a general questionnaire. Standing height was measured using a nonstretched tape fixed to the wall to the nearest 0.1 cm and weight by digital balance scale in light clothing to the nearest 0.1 kg. BMI was calculated using the following equation: BMI = weight (kg)/height^2^ (m). Using a tape measure, the circumference of the waist was measured from the midpoint of the lower rib and iliac crest. It should be noted that to decrease the error rate, all measurements were taken by the same person. Fat mass (FM), fat‐free mass (FFM), and body fat percent (BFP) were measured via bioelectric impedance analysis (InBody s10; Korea). An International Physical Activity Questionnaire (IPAQ) was used for pre‐ and poststudy measurements of physical activity levels. An experienced dietitian assessed dietary intake through a 24‐h recall questionnaire at the beginning and at the end of the study. Averaging food recalls were entered into Nutritionist IV as grams to obtain macro‐ and micronutrient composition.

### 2.4 Assessment of serum inflammatory and oxidative stress markers, leptin, and endothelin

The participant's blood samples were collected at the beginning and end of the intervention after 12‐h daytime fasting between 7:30 and 9:00 a.m. Afterward, the serums were allocated and stored at −80°C until the day of analysis. Serum IL‐6 and high‐sensitivity C‐reactive protein (hs‐CRP) concentrations were measured using an enzyme‐linked immunosorbent assay (ELISA) kit (Pars Azmoon Co., Tehran, Iran). Total antioxidant capacity (TAC) and malondialdehyde (MDA) contents were assessed by spectrophotometry using the standard kit (Pars Azmun Co, Tehran, Iran). Serum concentrations of leptin and endothelin were determined using a commercially available ELISA kit (Pars Azmoon Co., Tehran, Iran). It should be noted that our results were repeated three times on the samples. Also, between work runs, previous samples have been used for accuracy and precision of testing.

### 2.5 Statistical analyses

Data are presented as mean and standard deviation (SD). Normal distributions of continuous variables were checked by the Kolmogorov–Smirnov test. To compare means between the two groups, independent sample *t*‐test and Mann–Whitney *U* test were applied. To compare means within the two groups, paired *t*‐test or Wilcoxon signed‐rank tests were used. A chi‐square test was used to compare the distributions of categorical variables between the two groups. The postintervention values were compared using the analysis of covariance (ANCOVA) when baseline values were controlled for. For statistical analyses, IBM SPSS Statistics, version 24, was used, with a significance level of *p* < .05.

## 3 RESULTS

A total of 66 participants were enrolled in this study between June 2020 and September 2020. At the end of the trial, 63 subjects completed the study (Figure 1). Nutritional composition of cakes and treated acorn flour per 100 g is reported in Table 1. It should be noted that both cakes were well tolerated by all participants and there were no adverse reactions reported. Also, full compliance with interventions was evident by the fact that all packages were returned empty biweekly.

**TABLE 1:** Nutritional composition of cakes and treated acorn flour per 100 g.

Sample	Protein (w.p)	Pro NRV (w.p.)	Fat (w.p.)	Fiber (%)	Ash (w.p.)	Moisture (w.p.)	Total CHO (%)	Tannins (g)	Energy (kcal)
Acorn cake	5.6^a^	11.2	16.8	0.5	1.6	44.3	31.2	0.1	303.4
Control cake	7.3	14.5	15.0	Not detect	1.3	44.9	31.5	Not detected	295.6
Treated acorn flour	4.9	9.8	6.2	1.8	1.2	6.7	79.2	Not examined	399.9

Abbreviations: CHO, carbohydrate, Pro NR, protein nutrient reference value; w.p., weight percent.

^a^ Data are reported as mean.

Baseline characteristics of participants who completed the 8‐week intervention are presented in Table 2. Accordingly, we did not observe any significant difference between the two groups. Based on 3‐day dietary recalls and the IPAQ tool, no statistically significant difference was seen between the two groups regarding dietary intake and physical activity throughout the study (Table 3).

**TABLE 2:** Baseline participant characteristics.

Variables	PC (*n* = 33)	FC (*n* = 33)	*p*‐value
Age (years)*	42.4 ± 7.8	43.5 ± 7.3	.6
Sex			
Male	11	16	.2*
Female	22	17	
Calorie intake, kcal	2075.0 (1688.0, 2707.0)	1510.0 (1213.0, 1815.0)	.7
Physical activity (MET.min/week)	321.0 (150.0, 1110.0)	480.0 (270.0, 897.0)	.9
Height (cm)	165.7 ± 8.6	165.8 ± 10.5	.9
Weight (kg)	84.2 ± 11.1	82.0 ± 11.8	.4
BMI (kg/m^2^)	29.4 ± 4.1	29.3 ± 3.4	.9
WC (cm)	103.0 (99.0, 109.0)	99.0 (94.5, 105.0)	.1
Fat mass (kg)	30.9 ± 7.3	28.9 ± 6.5	.3
Body fat percent (%)	35.30 (31.3, 43.0)	36.1 (27.8, 40.0)	.4
Fat‐free mass (kg)	48.9 (44.2, 59.4)	48.3 (43.4, 58.0)	.7
Systolic blood pressure (mmHg)	136.3 ± 18.7	133.6 ± 16.5	.5
Diastolic blood pressure (mmHg)	88.0 (85.0, 95.0)	85.0 (80.0, 90.0)	.4
Fasting blood sugar (mg/dL)	97.9 ± 20.0	102.1 ± 18.3	.4
Insulin (mU/L)	18.4 ± 7.4	19.8 ± 7.8	.5
Triacylglycerol (mg/dL)	177.0 ± 8.8	173.8 ± 8.3	.9
Total cholesterol (mg/dL)	186.4 ± 30.2	200.0 ± 60.9	.3
Low‐density lipoprotein (mg/dL)	111.0 ± 29.3	113.3 ± 36.0	.8
High‐density lipoprotein (mg/dL)	41.7 ± 7.3	40.9 ± 7.0	.7
IL‐6 (pg/mL)	8.1 ± 4.2	7.8 ± 1.3	.7
hs‐CRP (mg/dL)	7.1 ± 2.0	7.1 ± 1.8	.9
MDA (mg/dL)	175.2 ± 73.3	177.6 ± 81.4	.9
TAC (mg/dL)	186.0 ± 25.6	196.4 ± 57.4	.5
Endothelin (mg/dL)	110.0 ± 33.6	111.6 ± 35.2	.7
Leptin (mg/dL)	41.5 ± 6.9	43.5 ± 7.3	.3
Abdominal obesity (men: ≥40 in., women: ≥35 in.)			.001
Yes	29 (87.9)	17 (51.5)	
No	4 (12.1)	16 (48.5)	
Hypertension (≥130/85 mmHg)			.8
Yes	13 (39.4)	12 (36.4)	
No	20 (60.6)	21 (63.6)	
Hypertriglyceridemia (≥150 mg/dL)			.8
Yes	14 (42.4)	13 (39.3)	
No	19 (57.6)	20 (60.6)	
Low−high‐density lipoprotein (Men: <40 mg/dL, Women: <50 mg/dL)			.2
Yes	20 (60.6)	25 (75.8)	
No	13 (39.4)	8 (24.2)	
Impaired glucose tolerance (≥100 mg/dL)			.1
Yes	9 (27.3)	16 (48.5)	
No	24 (72.7)	17 (51.5)	

*Note*: All outcomes reported as mean ± standard deviation or median (25th, 75th). *Independent sample *t*‐test or Mann–Whitney *U* test was used for comparison of quantitative variable and Chi‐square test or Fisher's exact test were used for comparison of qualitative variables.

Abbreviations: BMI, body mass index (kg/m^2^); hs‐CRP, high‐sensitive C‐reactive protein; IL‐6, interleukin 6; MDA, malondialdehyde; MET, metabolic equivalent; PC, placebo cake group; TAC, total antioxidant capacity; WC, waist circumference.

**TABLE 3:** Dietary intakes and physical activity of study participants throughout the study^a^.

	PC (*n* = 33)	FC (*n* = 33)	*p*‐value
	Mean ± SD	Mean ± SD	
Calorie intake, kcal/day			
Before	2075.0 (1688.0, 2707.0)	1510.0 (1213.0, 1815.0)	.7
After	1586.0 (1237.0, 1802.0)	1426.0 (1130.0, 1935.0)	.5
Carbohydrate intake, % of energy intake/day			
Before	37.3 ± 16.1	36.9 ± 10.7	.9
After	47.5 ± 16.8	51.1 ± 18.5	.4
Protein, % of energy intake/day			
Before	32.1 ± 7.4	36.9 ± 8.9	.4
After	23.6 ± 4.6	16.3 ± 4.6	.3
Fat, % of energy intake/day			
Before	30.6 ± 10.5	26.2 ± 6.6	.05
After	28.9 ± 10.4	32.6 ± 11.3	.2
Fiber intake, g			
Before	16.5 ± 6.6	18.6 ± 5.6	.6
After	23.5 ± 12.0	24.9 ± 11.4	.5
Physical activity (MET.min/week)			
Before	321.0 (150.0, 1110.0)	480.0 (270.0, 897.0)	.9
After	630.0 (275.6, 1171.5)	408.0 (255.0, 720.0)	.7

^a^ All outcomes reported as mean ± standard deviation. Independent sample *t*‐test or Mann–Whitney *U* test was used for comparison of quantitative variables.

Abbreviations: FC, functional acorn cake group; MET, metabolic equivalent; PC, placebo cake group.

There was a decrease in both groups' weight (kg), BMI (kg/m^2^), WC (cm), body fat percentage (%), and hip circumference (cm) at the end of the study compared with the baseline, (Table 4). No difference between groups in body weight, WC, body FM, and body fat percentage was observed (*p* > .05) (Table 4). After adjustment for baseline levels, consumption of FC compared to the PC resulted in a significant decrease in IL‐6 (changes from baseline: −1.7 ± 1.6 vs. −0.8 ± 1.7 mg/dL, *p* = .03) and hs‐CRP (changes from baseline: −2.3 ± 1.2 vs. −1.6 ± 1.7 mg/dL, *p* = .04) levels (Table 4). No differences were observed between groups with regard to MDA, TAC, endothelin, and leptin levels (*p* > .05).

**TABLE 4:** Body composition and metabolic measures at baseline and 8 weeks in overweight/obese adults with metabolic syndrome.

Parameters	PC (*n* = 32)				FC (*n* = 31)				Between groups		
	Baseline	End‐study	Change	*p*‐value^c^	Baseline	End‐study	Change	*p*‐value^c^	*p*‐value^b^	*p*‐value^a^	Mean difference
Weight (kg)	83.9 ± 12.2	79.9 ± 10.9	−4.2 ± 2.3	<.001	81.8 ± 10.8	76.8 ± 11.4	−5.1 ± 2.8	<.001	.1	.1	−0.9
BMI (kg/m^2^)	29.3 ± 4.6	28.3 ± 4.3	−1.1 ± 1.2	<.001	29.7 ± 3.9	27.8 ± 3.3	−1.4 ± 1.3	<.001	.3	.3	−0.3
WC (cm)	103.0 (99.0, 109.0)	99.5 (98.0, 108.7)	−4 (−4.4, −3.1)	<.001	99.0 (94.5, 105.0)	98.0 (91.0, 100.5)	−4.5 (−5, −3.0)	<.001	.1	–	−0.6
Fat mass (kg)	30.5 ± 5.3	28.8 ± 6.8	−1.7 ± 2.4	<.001	28.5 ± 4.5	27.1 ± 7.4	−2.8 ± 3.5	<.001	.1	.1	−1.1
Body fat percent (%)	35.30 (31.3, 43.0)	36.2 (28.6, 40.1)	−2.4 (−4.2, −0.6)	.01	36.1 (27.8, 40.0)	35.0 (27.8, 40.0)	−2.1 (−4.0, −0.8)	<.001	.2	–	−0.9
Fat‐free mass (kg)	48.9 (44.2, 59.4)	48.4 (43.6, 55.1)	−2.01 (−4.2, 0.2)	<.001	48.3 (43.4, 58.0)	45.0 (43.0, 58.4)	−1.1 (−3.2, 0.7)	.077	.2	–	+0.6
IL‐6 (pg/mL)	8.0 ± 4.5	7.3 ± 1.8	−0.8 ± 1.7	.01	7.3 ± 3.6	5.9 ± 1.6	−1.7 ± 1.3	<.001	.02	.03	−0.9
hs‐CRP (mg/dL)	7.5 ± 2.5	5.4 ± 1.3	−1.6 ± 1.7	.01	7.2 ± 3.8	4.7 ± 0.9	−2.3 ± 1.2	<.001	.04	.04	−0.7
MDA (mg/dL)	174.2 ± 77.1	147.0 ± 84.1	−27.2 ± 61.8	.01	178.6 ± 81.4	133.3 ± 54.5	−45.3 ± 51.0	<.001	.2	.2	−13.3
TAC (mg/dL)	188.0 ± 29.6	181.3 ± 30.6	−6.7 ± 37.0	.01	199.4 ± 57.4	177.3 ± 43.6	−22.1 ± 22.9	<.001	.04	.1	−15.4
Endothelin (mg/dL)	113.0 ± 28.3	104.5 ± 30.1	−8.5 ± 31.7	.01	113.6 ± 35.2	98.7 ± 29.2	−14.9 ± 18.7	<.001	.3	.3	−6.4
Leptin (mg/dL)	41.5 ± 6.9	43.3 ± 7.5	+1.7 ± 5.1	.1	40.5 ± 7.3	45.3 ± 8.4	+3.9 ± 4.7	<.001	.1	.1	+2.2

*Note*: The outcomes reported as mean ± standard deviation or median (25th, 75th).

Abbreviations: BMI, body mass index (kg/m^2^); FC, functional acorn cake group; hs‐CRP, high‐sensitive C‐reactive protein, IL‐6, Interleukin 6; MDA, malondialdehyde; PC, placebo cake group; TAC, total antioxidant capacity;WC, waist circumference.

^a^ ANCOVA adjusted for baseline value.

^b^ Difference between groups (independent sample *t*‐test or Mann–Whitney *U* test).

^c^ Difference from baseline (paired samples *t*‐test or Wilcoxon signed‐rank test).

## 4 DISCUSSION

The study has been conducted to investigate the effects of a prepared cake with acorn flour compared to a cake prepared with wheat flour on some biomarkers of obese and overweight patients with MetS. The results showed beneficial effects of FC on inflammatory markers (IL‐6 and hs‐CRP). However, it appeared that there were no significant favorable effects on serum oxidative stress marker (TAC), lipid peroxidation, leptin, or endothelin levels in the research. These findings suggest that food items containing natural elements can manage inflammation related to chronic diseases such as MetS.

In this study, in contrast to a cake made with wheat flour, a cake with acorn flour significantly reduced serum levels of inflammatory biomarkers. As far as we know limited human evidence is available that investigated the effects of acorn or acorn products on serum inflammatory markers. However, in experimental models of chronic disease, some species of Quercus were found to improve inflammatory markers. In a rat model of ulcerative colitis, the authors found that intervention with Quercus brantii gel significantly improved inflammatory markers such as TNF‐α, and IL‐6 (Alizade Naini et al., 2021). Acorn fruit's anti‐inflammatory effects may be due to its specific nutritional composition. For example, researchers found that ellagitannins from European oak bark, *Quercus petraea L*., reduced plasma CRP in rats fed a high‐carbohydrate, high‐fat diet (Panchal & Brown, 2013). The anti‐inflammatory effects of this compound, ellagitannins, have also been documented in other research (Larrosa et al., 2010). In addition, acorn is a rich source of oleic acid that has been shown to have anti‐inflammatory properties (Akcan et al., 2017). Moreover, acorn fruit's anti‐inflammation properties may be attributed to its high concentration of phenolic acids (gallic acid and ellagic acid) and flavonoids (naringin, quercetin, and catechin) (Alizade Naini et al., 2021). Gallic acid and phenolic compounds have been found to suppress IL‐6/p‐STAT3 (Y705) and p65‐NF‐κB activation and exert potentially clinically useful anti‐inflammatory effects (Pandurangan et al., 2015).

Our study found nonsignificant beneficial effects of acorn cake on markers of lipid peroxidation (MDA) and oxidative stress (TAC). Our results are in line with a trial that investigated the effects of acorn muffin consumption on complications of diabetes in patients with type 2 diabetes (Sasani et al., 2023). In that study also 8‐week intervention with acorn muffin did not improve the serum levels of MDA and TAC. However, the acorn constitutes have shown significant benefits in the reduction of lipid peroxidation and oxidative stress in experimental studies. Accordingly, a study by Alizade Naini et al. indicated that Quercus brantii gel significantly improved lipid peroxidation (MDA) and oxidative stress (superoxide dismutase) markers in rat model of ulcerative colitis (Alizade Naini et al., 2021). In another study, ellagitannins derived from *Quercus petraea L*. significantly improved plasma MDA and glutathione peroxidase activity in rats fed with carbohydrate, high‐fat diet (Panchal & Brown, 2013). Further, Dogan et al. reported that lyophilized extracts from acorns, *Quercus brantii Lindl*., significantly reduced MDA and significantly increased antioxidant defense in STZ‐induced diabetic rats (Dogan et al., 2015). The significant versus nonsignificant results from experimental and human studies might be because of some facts. First of all, researchers have studied specific components of acorn fruit, such as ellagitannins and lyophilized extracts from acorns, which are usually concentrated in amounts over what is normally consumed by humans. Accordingly, if the period of the research or the dose of the intervention is increased, the results of human studies may become meaningful. In addition, the antioxidant activity of acorn extract is attributed to water‐soluble components and is also dependent on its concentration (Rakić et al., 2006). So, there is a possibility that our results could be linked to the loss of some antioxidant compound during the treatment of acorns or how much treated flour was used. Moreover, management of potential confounders in human studies usually is more difficult than in experimental studies which may affect the results. Thus, the antioxidant capacity of acorn or acorn products needs to be investigated in future research.

Finally, this study failed to find any favorable effects of acorn‐based cake on serum levels of leptin and endothelin. Based on the evidence, there is a positive link between serum leptin and the risk of MetS in both obese and nonobese populations (Esteghamati et al., 2011; Yun et al., 2010). Therefore, serum leptin levels may be a predictor of MetS (Ghadge & Khaire, 2019). Regarding serum levels of endothelin, it has been documented that MetS is associated with higher endothelin‐1 vasoconstrictor tone in overweight/ obese adults (Rocha et al., 2014). This condition was found to be associated with higher blood pressure in adults (Yau, 2013) or elderly (Chung, 2015) populations. As far as we know, there is no study investigating the effects of acorn or acorn products in the improvement of such hormones. However, in an animal study, rats supplemented with rutin, a highly concentrated component of acorn fruit, experienced a significant decrease in leptin levels (Hsu et al., 2009). In addition, researchers found that phenolic compounds, abundant in acorn fruit, reduced serum leptin levels significantly in obese rats (Saeed et al., 2018). Regarding endothelin, a randomized, placebo‐controlled, crossover study showed that supplementation with 200‐mg dietary flavonoids (quercetin, epicatechin, and/or epigallocatechin gallate) significantly improved serum levels of endothelin‐1 (Loke et al., 2008). In addition, acorn fruit mainly contains epicatechin, which is found to lower plasma endothelin‐1 levels in hypertension rats, preventing hypertension as a result (Gómez‐Guzmán et al., 2012). In general, as mentioned above, possible favorable effects of major components of acorn fruit have been found in previous research which are inconsistent with ours because of the following reasons: (A) in our study, weight loss was not different between groups despite the energy‐restricted diets, indicating that caloric restriction had a greater impact on weight loss than functional cake. As a result of this weight loss, obesity‐related complications could be reduced, leading to improved health. However, it seems this weight change was not enough to change serum levels of leptin or endothelin. (B) lower study duration can also be another reason for these nonsignificant findings. (C) lastly, researchers have studied specific components of acorn fruit, such as ellagitannins and lyophilized extracts from acorns, which are usually concentrated in amounts over what is normally consumed by humans. So, Acorns and acorn‐derived products should be investigated further in the future to identify possible beneficial effects on serum hormone regulation as well as the pathway by which they act.

This study has some strengths. A valuable functional food was introduced with high health benefits in this study, which is also reflected by the high compliance rate of the participants in the study. Our study had a randomized design which also increased the validity of the study. Nevertheless, similar to any other research, our study also had some limitations. We were unable to assess whether the acorn‐based cake had a lasting effect on other cardiometabolic parameters such as body composition because of the short duration of the intervention. This also has been found in another clinical trial that acorn muffin consumption marginally improved glycated hemoglobin, triglyceride, and high‐density lipoprotein after 8 weeks of intervention (Sasani et al., 2023). This means the short duration of the studies might be a reason for the moderate beneficial effects of acorn‐based products on cardio‐metabolic risk factors. In this study, we could not characterize the main components of acorns due to the limited available techniques and their cost. The study was calorie‐restricted by design, so it is unknown whether acorn‐based cake can act as a treatment for MetS. Therefore, it needs further investigation whether receiving this product can be beneficial in treating MetS.

In conclusion, this study demonstrated that acorn‐based cake could improve inflammation as an adjunct to an energy‐restricted diet in overweight and obese patients with MetS. However, it is not clear whether acorn‐based cake can be used to prevent or treat MetS because of indecisive findings regarding its ability to manage oxidative stress and serum hormones. Further studies will be needed to determine whether or not it can be used as a preventative strategy or adjunct treatment.

## AUTHOR CONTRIBUTIONS

H. S.‐D.: Data collection, sample analysis, writing—original draft. S.B. and M.M.‐S.: conceptualization, writing—review and editing. M.A.: Methodology, investigation, and writing—review and editing.

## FUNDING INFORMATION

This study was conducted with financial support that was provided by the Shiraz University (grant number: 1400‐03‐21).

## ETHICS STATEMENT

The study protocol was approved by the ethics scientific committee of the Shiraz University (ethical code number: IR.us.rec.1402.009) and informed consent was obtained from all individual participants included in the study.

## CONSENT

The study's authors affirm that there were no financial or commercial ties that might be viewed as having a potential conflict of interest.

## Data Availability

The data that support the findings of this study are available on request from the corresponding author. The data are not publicly available due to privacy or ethical restrictions.
